# Secondary hypogammaglobulinemia and lymphocytopenia in patients with inflammatory neurological diseases on anti-CD20 therapy: risk of infection and infection-related mortality

**DOI:** 10.1007/s10072-025-08329-x

**Published:** 2025-07-05

**Authors:** Bedriye Karaman, Göktuğ Dinçer, Rasim Tunçel, Ozgul Ekmekci, Nur Yüceyar

**Affiliations:** Faculty of Medicine, Department of Neurology, Ege University, Bornova, Izmir, 35100 Turkey

**Keywords:** Ocrelizumab, Rituximab, Infection, Hypogammaglobulinemia, Lymphopenia, Mortality

## Abstract

Although anti-CD20 treatments are effective in inflammatory neurological diseases, they have some risks, especially infections. Determining the predictor factors of infection helps risk management in patients receiving anti-CD20 treatments. The effect of ocrelizumab (OCR) and rituximab (RTX) associated hypogammaglobulinemia (HGG) and lymphopenia on infection risk is controversial. The aim of this study to evaluate relationship between HGG and lymphocytopenia and infection risk and infection-related mortality in patients under RTX and OCR treatments and also compare these parameters between two agents. In our findings no relationship was found between HGG and infection risk in patients receiving OCR and RTX. In the RTX group, a significant relationship was detected between lymphocytopenia and severe infection. And, all three patients with infection-related mortality under rituximab treatment had HGG. No infection-related mortality was happened in OCR treatment. While lymphocytopenia and HGG were not detected as significant risk factors for infection, older age, female gender and > 4 EDSS score were determined as risk factors for infection in patients receiving OCR treatment. Identifying factors predicting infection risk may provide better risk management in patients receiving anti-CD20 therapy.

## Introduction

Anti-CD20 therapies, such as rituximab (RTX) and ocrelizumab (OCR), are commonly used to treat neurological inflammatory diseases. They target and deplete CD20 + B cells, which include mature and memory B cells that are precursors to antibody-producing plasma cells. This depletion reduces immunoglobulin (IgG, IgM, IgA) levels, potentially compromising the immune response to pathogens. These B cell depleting therapies can lead to secondary hypogammaglobulinemia (HGG), which may increase the risk of infections [1]. Lymphocytopenia occurs in approximately 40% of patients receiving anti-CD20 therapy and has been associated with a higher incidence of bacterial and viral infections [2, 3].

Rituximab is a chimeric anti-CD20 monoclonal antibody used in the treatment of various neurological disorders including multiple sclerosis (MS), neuromyelitis optica spectrum disorders (NMOSD), myasthenia gravis (MG), chronic inflammatory demyelinating polyneuropathy (CIDP), autoimmune encephalitis (AIE), paraneoplastic syndromes (PNS), and thyroid ophthalmopathy. Although secondary HGG developing under RTX treatment has been shown in many studies, it is controversial whether this increases the risk of infection [4–6]. In a study evaluating 169 NMOSD patients who received an average of 10 doses of RTX, it was reported that 41% of the patients developed HGG, but this did not increase the infection risk [4]. In contrast, a prospective study has shown that patients treated with RTX often have decreased IgG levels, increasing the risk of symptomatic infection in vulnerable patients [7].

Ocrelizumab is a humanized anti-CD20 monoclonal antibody that is highly effective for relapsing-remitting MS and is the only approved treatment for primary progressive MS. In ORATORIO study comparing OCR with placebo in primary progressive MS, OCR was associated with an increased risk of infections, including upper respiratory tract infection and oral Herpes [8]. A Canadian cohort of 266 OCR-treated MS patients showed no association between HGG and serious infection [9]. In a cohort study of MS patients treated with OCR from Australia, higher serum IgA, IgG levels and older age were associated with a reduced risk of infection [10].

Studies evaluating the causal relationship between infection and HGG or lymphocytopenia in patients receiving anti-CD-20 therapy that mentioned above had conflicting results. This study examines the relationship between HGG and lymphocytopenia with infection risk and infection-related mortality in patients with inflammatory neurological disease who have undergone anti-CD20 therapies. And we have also compared presence of HGG and lymphocytopenia and infection risk and infection-related mortality between OCR and RTX treatments” as “We also compared the presence of HGG and lymphocytopenia as well as the risk of infection and infection-related mortality between the OCR and RTX treatments.

## Materials and methods

All patients with neurological disorders who were treated with either OCR or RTX in Ege University Hospital Neurology Department were retrospectively reviewed for inclusion in the study. Patients over 18 years of age, diagnosed with MS, NMOSD, CIDP, AIE, PNS, MG, and receiving RTX or OCR, were selected. Patients were excluded from the study if they did not receive at least two treatment cycles, if IgG and lymphocyte levels were not adequately measured before and after treatment, if they had a major confounding hematologic disorder, or if they were using another treatment that could cause HGG, such as carbamazepine.

A serum IgG level below 600 mg/dL was defined as HGG [5] and a lymphocyte count below 1000/mcL was defined as lymphocytopenia. Lymphocytopenia were further divided into grade 1 (800–999/mcL), grade 2 (799 − 500/mcL), grade 3 (499 − 200/mcL), and grade 4 (< 200/mcL) [11]. IgG, IgA, IgM, neutrophil and lymphocyte levels before and throughout treatment were also noted to determine minimum levels during treatment. For all patients, baseline characteristics were collected including age, gender, diagnosis, time on therapy, whether presence of previous treatment and type of B-cell depleting agent. Infectious complications during treatment course were noted and divided into mild (not requiring hospitalization or IV therapy) and serious (requiring hospitalization or IV therapy.). For MS patients under OCR therapy, EDSS was calculated and the current MS phenotype [12] (relapsing remitting, primary progressive or secondary progressive) was determined.

Statistical analyses were conducted, including the t-test or Mann-Whitney U test for comparing group means, the chi-squared test or Fisher’s exact test for categorical variables, Pearson correlation for linear relationships, and logistic regression to identify significant predictors of infection. *P*≤ 0.05 was considered statistically significant. The study protocol (n.24-12T/45) was approved by the Ethical Committee of Ege University.

## Results

A total of 257 patients under OCR therapy and 52 patients under RTX therapy were evaluated for inclusion in the study. After excluding patients according to the criteria mentioned above 148 patients receiving OCR and 43 patients receiving RTX were included in the statistical analysis. Baseline demographics of each group are shown in Table 1.

**Table 1:** Baseline characteristics of patients

	All patients (*n* = 191)	Ocrelizumab (*n* = 148)	Rituximab (*n* = 43)
Age (years)^1^	50.8 ± 11.1	51.8 ± 9.6	47.4 ± 14.8
Female, n (%)^2^	121 (%63,4)	89 (%60.1)	32 (%74.4)
Diagnosis, n (%)	MS 152 (%79.6) NMOSD 19 (%9.9) MG 10 (%5.2) PNS 5 (%2.6) CIDP 3 (%1.6) AIE 2 (%1.0)	RRMS 42 (%28.4) SPMS 68 (%45.9) PPMS 38 (%25.7)	NMOSD 19 (%44.2) MG 10 (%23.3) PNS 5 (%11.6) MS 4 (%9.3) CIDP 3 (%7) AIE 2 (%4.7)
Treatment Cycles, median, min-max	MS 5.0 (2–10) NMOSD 3.0 (2–8) MG 2.5 (2–9) PNS 2.0 (2–8) CIDP 2.0 (2–3) AIE 3.0 (2–4)	5 (2–10)	3 (2–10)
EDSS, median, min-max		4.0 (1.0–8.0)	
No previous therapy, n (%)	38 (%19.9)	29 (%19.6)	9 (%20.9)
Infectious complications, n (%)	86 (%45.0)	66 (%44.6)	20 (%46.5)
HGG, n (%)	20 (%10.5)	14 (%9.5)	6 (%13.9)
Lymphocytopenia, n (%)	55 (%28.8)	45 (%30.4)	10 (%23.3)

1 and 2. Age and sex were not significantly different between two groups. NMOSD: neuromyelitis optica spectrum disorder, MG: myasthenia gravis, PNS: paraneoplastic syndrome, CIDP: chronic immune demyelinating polyneuropathy, AIE: autoimmune encephalitis, HGG: hypogammaglobulinemia

In this cohort, the incidence of infection was 45.0% in all patients receiving anti-CD20 treatment. Among 148 patients receiving OCR, 66 (44.6%) experienced an infection during therapy. Of these infections, 8 (5.4%) were considered serious, requiring IV therapy or hospitalization. Urinary tract infections were the most common, affecting 62 patients (41.9%). Fewer patients had respiratory tract infections (5 patients, 3.4%) or skin infections (5 patients, 3.4%). No infection-related mortality occured in OCR treatment. On the other hand, 20 (46.5%) of 43 patients receiving RTX experienced infection. The most common type of infection was urinary tract infection (9 patients, 21%), followed by respiratory tract infections (7 patients, 16%), skin infections (2 patients, 4.7%) and gastrointestinal infections (2 patients, 4.7%). Five (11.6%) of these infections were considered serious and two (4.7%) infection-related mortality were observed in patients receiving RTX. Both of these patients had HGG, and one had lymphocytopenia. In the RTX group, patients with and without infection did not differ by age and sex. However, in the OCR group, patients with infection had a higher age, female dominance, and a higher disability compared to those without infection. A logistic regression model confirmed female sex (exp(B) = 6.522 [2.894–14.698] *p* < 0.001), age of at least 60 (exp(B) = 3.037 [1.219–7.567] *p* = 0.017) and EDSS of at least 4.5 (exp(B) = 2.133 [1.002–4.539] *p* = 0.046) as independent risk factors for infection in patients receiving OCR. In our sample, no other variable demonstrated significant potential as a risk factor for infection in patients receiving RTX and OCR. Relevant information is given in Table 2.

**Table 2:** Comparison of patients with and without infection

	Ocrelizumab (*n* = 148)			Rituximab (*n* = 43)		
	Patients with infection (*n* = 66)	Patients with no infection (*n* = 82)	*p*	Patients with infection (*n* = 20)	Patients with no infection (*n* = 23)	*p*
Age	**53.9 ± 9.9**	**50.0 ± 9.0**	**0.013**	48.4 ± 13.5	46.5 ± 16.1	0.675
Female	**55 [%83.3]**	**34 [%41.5]**	**< 0.001**	16 [%80.0]	16 [%69.6]	0.434
Min IgG	9.2 ± 2.4	8.9 ± 2.4	0.408	8.4 ± 3.3	9.0 ± 2.4	0.519
Min IgA	1.72 ± 0.83	1.67 ± 0.90	0.436	1.5 ± 0.7	1.7 ± 0.8	0.404
Min IgM	0.77 ± 0.53	0.65 ± 0.57	0.504	0.79 ± 0.51	0.63 ± 0.32	0.443
Min Lym.	1.20 ± 0.44	1.31 ± 0.50	0.203	1.45 ± 0.69	1.71 + 0.74	0.233
Min Neu.	3.57 ± 1.32	3.74 ± 1.34	0.577	4.95 ± 1.90	3.92 ± 2.35	0.761
IgG < 6 mg/dL	6 [%9.1]	8 [%9.8]	0.891	5 [%25.0]	1 [%4.3]	0.650
Lym. < 1000/mcL	22 [%33.3]	23 [%28.0]	0.487	6 [%30.0]	4 [%17.4]	0.269
Lym < 500/mcL	3 [%4.5]	4 [%4.9]	0,619	1 [%0.5]	0 [%0.0]	0.465
Cycle count, n, median [min-max]	5 [2–10]	4.5 [2–10]	0.305	2 [2–10]	2 [2–9]	0.483
EDSS ≥ 4	**49 [%74.2]**	**47 [%57.3]**	**0.032**			

The chi-squared test, Fisher’s exact test, t-test or Mann-Whitney U test were utilized when appropriate. Statistically significant variables are indicated in bold

A comparative analysis of patients with and without serious infections in RTX and OCR groups was conducted. The analysis showed no significant differences in age, sex or HGG (*p* > 0.05). However, lymphocytopenia was associated with a higher serious infection risk in RTX patients. Of the 5 patients with serious infection, 4 (80.0%) had lymphocytopenia, whereas only 6 (15.8%) of the 38 patients without serious infections had lymphocytopenia (*p* = 0.007). In OCR patients, there was no association between lymphocytopenia and serious infections (*p* = 0.540).

28.8% of patients on anti-CD20 treatment had lymphocytopenia. No significant relationship was detected between minimum lymphocyte count, presence of lymphocytopenia and infection in both OCR and RTX groups. Patients with lymphocytopenia (*n* = 10) compared to those who did not develop lymphocytopenia (*n* = 33), were not significantly different regarding age or sex (age: 53.9 ± 20.8 vs. 45.4 ± 12.3, *p* = 0.244, sex: 5 [50.0%] females vs. 27 [81.9%], *p* = 0.580) in RTX group. Received RTX cycle count was not related to an increase in lymphocytopenia (*p* = 0.19). In lymphocytopenic patients (*n* = 45) compared to non-lymphocytopenic patients (*n* = 103) no significant differences were observed for age, sex or EDSS (age: 52.2 ± 9.3 vs. 51.6 ± 9.8, *p* = 0.726, sex: 32 [71.1%] females vs. 57 [55.3%] females, *p* = 0.071, EDSS ≥ 4: 28 [62.2%] vs. 68 [66.0%], *p* = 0.656) in OCR group. Received OCR cycle count was not related to an increase lymphocytopenia (*p* = 0.353).

Hypogammaglobulinemia rate was 10.5% in our cohort. In both OCR and RTX groups, no significant relationship was found between minimum levels of IgG, IgA, IgM, presence of HGG and infection. In the RTX group patients who developed HGG (*n* = 6) did not differ from those who did not develop HGG (*n* = 37); in age or sex (age: 42.9 ± 16 vs. 48.1 ± 14.8, *p* = 0.429; sex: 4 [66.7%] females vs. 28 [75.7%] females, *p* = 0.488). Presence of HGG was not related with presence of lymphocytopenia (*p* = 0.127) in the RTX group. Minimum IgG and lymphocyte levels were also not correlated (*p* = 0.120). Received RTX cycle count was not related to an increase in HGG (*p* = 0.39). Among the patients receiving OCR, similar to the RTX group, those who developed HGG (*n* = 14) were not significantly different compared to those without HGG (*n* = 134) by sex (7 [50.0%] females vs. 82 [61.2%] females, *p* = 0.416) and did not have a higher proportion of high EDSS patients (EDSS ≥ 4, 10 [71.4%] vs. 86 [64.2%], *p* = 0.412). However, hypogammaglobulinemic patients were significantly older than those without HGG in OCR group (58.9 ± 7.5 vs. 51.0 ± 9.5, *p* = 0.003). Presence of HGG was not related with presence of lymphocytopenia (*p* = 0.572) and minimum IgG and lymphocyte levels did not correlate (*p* = 0.690) with each other. Received OCR cycle count was not related to an increase HGG (*p* = 0.863).

In comparative analysis of OCR and RTX groups, patients receiving OCR therapy (*n* = 148) did not significantly differ from those on RTX therapy (*n* = 43) in the frequencies of HGG (14 [9.5%] vs. 6 [14%], *p* = 0.277), lymphocytopenia (45 [30.4%] vs. 10 [23.3%], *p* = 0.362), or infections (66 [44.6%] vs. 20 [46.5%], *p* = 0.824). Minimum IgG levels were also similar between the two groups (9.06 ± 2.38 in OCR group vs. 8.70 ± 2.84 in RTX group, *p* = 0.407). However, patients on OCR had significantly lower minimum lymphocyte levels (median [min-max]; 1.22 [0.33–3.06] vs. 1.56 [0.32–3.13], *p* = 0.011).

## Discussion

This study aims to investigate the relationship between the development of HGG and lymphocytopenia and the risk of infection and infection-related mortality in patients under anti-CD20 treatment, as well as to compare OCR and RTX in this regard. Decrease in serum immunoglobulin G levels below the lower limit of normal is a well-known complication of anti-CD20 treatments [13]. Clinical trials suggest that not all anti-CD20 therapies affect IgG levels equally. For example, RTX and OCR have been shown to decrease IgG levels more than ofatumumab (OFA) and ublituximab [13]. There are conflicting results regarding the HGG complication of RTX, which is widely used in the treatment of many inflammatory neurological diseases, and OCR, which is used in almost every clinical form of MS. In our cohort 9.5% of patients developed HGG under OCR treatment and 14% of patients developed HGG under RTX treatment with no significant difference between two agents. In a recently published multicentric study, HGG was found more frequently in RTX than in OCR-treated patients [14]. In a meta-analysis evaluating over 12,000 patients, the frequency of HGG was highest in RTX with 18%, followed by OCR with 11% and 2% in OFA [15]. The frequency of HGG in RTX and OCR in this meta-analysis was found similar to the rates in our results.

In the recent study, lymphocytopenia rate was 28.8% in the entire cohort, 30.4% and 23.3% in the OCR and RTX groups, respectively. In another study, lymphocytopenia rate was 15.6% and no significant difference was found between anti-CD-20 treatments [16]. Lymphocytopenia was reported in 20.7% of RRMS patients with OCR treatment in OPERA I and II phase 3 trials [17]. In another cohort lymphocytopenia was found in 20% of patients taking OCR [18]. In a real-world study investigating the infection risk of long-term anti-CD20 therapy, lymphocytopenia was not associated with an increased risk of infection [19]. However, there are also studies indicating that lymphocytopenia increases the risk of infection [2, 3]. Minimum lymphocyte counts ​​were significantly lower in patients receiving OCR than in patients receiving RTX in our results. However, there was no significant association between lymphocytopenia and risk of infection/serious infection in patients receiving OCR, whereas the risk of serious infection was significantly higher in lymphopenic patients receiving RTX. One of two patients in RTX group who died because of infection had lymphocytopenia.

Increased infection risk is a well defined complication of anti-CD20 treatments from both clinical trials and real-life data [3, 8]. Our study reports 44.6% and 46.5% infection rate respectively among patients receiving OCR and RTX. Habek et al. showed that 58.7% of patients treated with OCR experienced infection during treatment, higher than our findings [20]. In a multicentric study, there was no significant difference found in proportion of patients who had infections between the two drugs as 38% patients on OCR compared to 39% patients on RTX developed infections [14]. These rates seem higher than previously reported meta-analysis (11% infection rate was reported by Elgenidy et al.), but they represent all infections, not only serious infections. Serious infection occurred 5.4% of patient receiving OCR treatment and 11.6% of patients receiving RTX treatment in our cohort. Similar serious infection rate was reported in OCR-treated MS patients in a real-world cohort [9].

Many studies have reported that anti-CD20 therapy associated HGG may increase the risk of infection [21]. Smolik et al. showed that severe HGG (< 500 mg/dl) was associated with severe infection in MS patients on anti-CD20 agents [14]. Saidha et al. conducted a systematic review of HGG in MS patients who was treated with anti-CD20 drugs and documented that decreasing IgG levels have been correlated with increased infection risk over time [22]. Our results showed that neither infection nor severe infection was associated with HGG in both OCR and RTX groups. Real-life data from NMOSD patients on 14 years of RTX treatment showed that approximately one-third of patients developed infections, 8% had serious infections, but there was no association between the risk of infection and HGG [4]. Infection-related mortality of anti-CD20 treatments is less investigated issue in previous studies. Two of 43 (4.7%) patients who had HGG in RTX group died because of serious infection in our cohort. Barmettler et al. reported that development of serious infection and HGG were significantly associated increased mortality in patients after receiving RTX [6].

Studies investigating infection risk in anti CD-20 treatment, various predictors have been revealed such as HGG, disease phenotype, lymphocytopenia, older age and duration of therapy [1, 19, 23]. Our findings showed that the predictors for infection in patients receiving OCR were female gender, age over 60, and EDSS of at least 4.5. However, no predictor factor for infection in patients receiving RTX was revealed.

Limitations of our study include single-center, retrospective design and low sample size and heterogeneity in the RTX group.

According to our findings in this cohort, no relationship was found between infection risk and HGG. In the RTX group, a significant relationship was found between lymphocytopenia and serious infection. In patients under OCR therapy, lymphocytopenia and HGG were not detected as significant risk factors for the development of infection, while older age, female gender and having high EDSS score were identified as risk factors for infectious complications. No infectious complication resulted in mortality in OCR group, however, two infection-related mortality were observed in patients receiving RTX who had also HGG. The predictors of infection risk in patients receiving anti-CD20 therapy highlights the need for careful monitoring and individualized management. By identifying and managing these risks proactively, healthcare providers can help optimize the therapeutic benefits of anti-CD20 therapies while minimizing adverse outcomes and mortality.

## References

[1] Mears V, Jakubecz C, Seeco C, Woodson S, Serra A, Abboud H (2023) Predictors of hypogammaglobulinemia and serious infections among patients receiving Ocrelizumab or rituximab for treatment of MS and NMOSD. J Neuroimmunol 377:578066 36917920 10.1016/j.jneuroim.2023.578066

[2] Zappulo E, Buonomo AR, Saccà F, Russo CV, Scotto R, Scalia G, Nozzolillo A, Lanzillo R, Tosone G, Gentile I (2019) Incidence and predictive risk factors of infective events in patients with multiple sclerosis treated with agents targeting CD20 and CD52 surface antigens. Open Forum Infect Dis 6(11):ofz445 31723572 10.1093/ofid/ofz445 PMC6837838

[3] Oksbjerg NR, Nielsen SD, Blinkenberg M, Magyari M, Sellebjerg F (2021) Anti-CD20 antibody therapy and risk of infection in patients with demyelinating diseases. Mult Scler Relat Disord 52:102988 33979772 10.1016/j.msard.2021.102988

[4] Kim SH, Park NY, Kim KH, Hyun JW, Kim HJ (2022) Rituximab-Induced hypogammaglobulinemia and risk of infection in neuromyelitis Optica spectrum disorders: A 14-Year Real-Life experience. Neurol Neuroimmunol Neuroinflamm 9(5) 10.1212/NXI.0000000000001179 PMC9296048 35853752

[5] Tallantyre EC, Whittam DH, Jolles S, Paling D, Constantinesecu C, Robertson NP, Jacob A (2018) Secondary antibody deficiency: a complication of anti-CD20 therapy for neuroinflammation. J Neurol 265(5):1115–1122 29511864 10.1007/s00415-018-8812-0 PMC5937879

[6] Barmettler S, Ong MS, Farmer JR, Choi H, Walter J (2018) Association of Immunoglobulin levels, infectious risk, and mortality with rituximab and hypogammaglobulinemia. JAMA Netw Open 1(7):e184169 30646343 10.1001/jamanetworkopen.2018.4169 PMC6324375

[7] Avouac A, Maarouf A, Stellmann JP, Rico A, Boutiere C, Demortiere S, Marignier R, Pelletier J, Audoin B (2021) Rituximab-Induced hypogammaglobulinemia and infections in AQP4 and MOG Antibody-Associated diseases. Neurol Neuroimmunol Neuroinflamm 8(3) 10.1212/NXI.0000000000000977 PMC8054961 33722933

[8] Montalban X, Hauser SL, Kappos L, Arnold DL, Bar-Or A, Comi G, de Seze J, Giovannoni G, Hartung HP, Hemmer B et al (2017) Ocrelizumab versus placebo in primary progressive multiple sclerosis. N Engl J Med 376(3):209–220 28002688 10.1056/NEJMoa1606468

[9] Nobile S, Beauchemin P Hypogammaglobulinemia and infection risk in an Ocrelizumab-treated multiple sclerosis cohort. Can J Neurol Sci 2024:1–8 10.1017/cjn.2024.21 38343112

[10] Seery N, Sharmin S, Li V, Nguyen AL, Meaton C, Atvars R, Taylor N, Tunnell K, Carey J, Marriott MP et al (2021) Predicting infection risk in multiple sclerosis patients treated with ocrelizumab: A retrospective cohort study. CNS Drugs 35(8):907–918 33847902 10.1007/s40263-021-00810-3 PMC8042832

[11] Dinoto A, Sartori A, Cheli M, Pasquin F, Baldini S, Bratina A, Bosco A, Manganotti P (2022) Lymphocytopenia during treatment with dimethyl fumarate in patients with multiple sclerosis: prevalence, predicting factors and clinical outcomes. Mult Scler Relat Disord 57:103357 35158466 10.1016/j.msard.2021.103357

[12] Klineova S, Lublin FD (2018) Clinical course of multiple sclerosis. Cold Spring Harb Perspect Med 8(9) 10.1101/cshperspect.a028928 PMC6120692 29358317

[13] Alvarez E, Longbrake EE, Rammohan KW, Stankiewicz J, Hersh CM (2023) Secondary hypogammaglobulinemia in patients with multiple sclerosis on anti-CD20 therapy: pathogenesis, risk of infection, and disease management. Mult Scler Relat Disord 79:105009 37783194 10.1016/j.msard.2023.105009

[14] Smolik K, Camilli F, Panzera I, Fiore A, Franceschini A, Foschi M, Surcinelli A, Pesci I, Ferri C, Bazzurri V et al (2024) Hypogammaglobulinemia and severe infections in multiple sclerosis patients on anti-CD20 agents: A multicentre study. Mult Scler Relat Disord 93:106191 39616774 10.1016/j.msard.2024.106191

[15] Elgenidy A, Abdelhalim NN, Al-Kurdi MA, Mohamed LA, Ghoneim MM, Fathy AW, Hassaan HK, Anan A, Alomari O (2024) Hypogammaglobulinemia and infections in patients with multiple sclerosis treated with anti-CD20 treatments: a systematic review and meta-analysis of 19,139 multiple sclerosis patients. Front Neurol 15:1380654 38699050 10.3389/fneur.2024.1380654 PMC11063306

[16] Fernandes AA, Neves AL, Ferro D, Seabra M, Mendonca T, Dos Reis RS, Sa MJ, Guimaraes J, Abreu P (2024) Clinical and analytical monitoring of patients with multiple sclerosis on anti-CD20 therapeutics: a real-world safety profile study. Front Neurol 15:1500763 39835156 10.3389/fneur.2024.1500763 PMC11743370

[17] Schweitzer F, Laurent S, Fink GR, Barnett MH, Hartung HP, Warnke C (2021) Effects of disease-modifying therapy on peripheral leukocytes in patients with multiple sclerosis. J Neurol 268(7):2379–2389 32036423 10.1007/s00415-019-09690-6 PMC8217029

[18] Abbadessa G, Maida E, Miele G, Lavorgna L, Marfia GA, Valentino P, De Martino A, Cavalla P, Bonavita S (2022) Lymphocytopenia in multiple sclerosis patients treated with Ocrelizumab is associated with an effect on CD8 T cells. Mult Scler Relat Disord 60:103740 35305426 10.1016/j.msard.2022.103740

[19] Peters J, Longbrake EE (2022) Infection risk in a real-world cohort of patients treated with long-term B-cell depletion for autoimmune neurologic disease. Mult Scler Relat Disord 68:104400 36544307 10.1016/j.msard.2022.104400 PMC10075342

[20] Habek M, Piskač D, Gabelić T, Barun B, Adamec I, Krbot Skorić M (2022) Hypogammaglobulinemia, infections and COVID-19 in people with multiple sclerosis treated with Ocrelizumab. Mult Scler Relat Disord 62:103798 35429819 10.1016/j.msard.2022.103798 PMC8994678

[21] Hauser SL, Cross AH, Winthrop K, Wiendl H, Nicholas J, Meuth SG, Giacomini PS, Saccà F, Mancione L, Zielman R et al (2022) Safety experience with continued exposure to ofatumumab in patients with relapsing forms of multiple sclerosis for up to 3.5 years. Mult Scler 28(10):1576–1590 35229668 10.1177/13524585221079731 PMC9330270

[22] Saidha S, Bell J, Harold S, Belisario JM, Hawe E, Shao Q, Wyse K, Maiese EM (2023) Systematic literature review of Immunoglobulin trends for anti-CD20 monoclonal antibodies in multiple sclerosis. Neurol Sci 44(5):1515–1532 36648561 10.1007/s10072-022-06582-y PMC9843103

[23] Smolik K, Camilli F, Panzera I, Fiore A, Franceschini A, Foschi M, Surcinelli A, Pesci I, Ferri C, Bazzurri V et al (2025) Hypogammaglobulinemia and severe infections in multiple sclerosis patients on anti-CD20 agents: A multicentre study. Mult Scler Relat Disord 93:106191 39616774 10.1016/j.msard.2024.106191

